# Introduction of the DiaGene study: clinical characteristics, pathophysiology and determinants of vascular complications of type 2 diabetes

**DOI:** 10.1186/s13098-017-0245-x

**Published:** 2017-06-19

**Authors:** Thijs T. W. van Herpt, Roosmarijn F. H. Lemmers, Mandy van Hoek, Janneke G. Langendonk, Ronald J. Erdtsieck, Bert Bravenboer, Annelies Lucas, Monique T. Mulder, Harm R. Haak, Aloysius G. Lieverse, Eric J. G. Sijbrands

**Affiliations:** 1 Department of Internal Medicine, Erasmus Medical Center, P. O. Box 2040, 3000 CA Rotterdam, The Netherlands; 2 Department of Internal Medicine, Máxima Medical Center, Ds Th Fliednerstraat 1, 5631 BM Eindhoven, The Netherlands; 3 Department of Internal Medicine, Catharina Hospital, Michelangelolaan 2, 5623 EJ Eindhoven, The Netherlands; 4 Center for Primary Care Diagnostics, Boschdijk 1119, 5626 AG Eindhoven, The Netherlands; 5 Department of Internal Medicine, Division of General Internal Medicine, Maastricht University Medical Center+, Maastricht, The Netherlands; 6 Maastricht University, CAPHRI School for Public Health and Primary Care, Ageing and Long-Term Care, Maastricht, The Netherlands; 7 Department of Endocrinology, University Hospital Brussels, Laarbeeklaan 101, 1090 Brussels, Belgium

**Keywords:** Type 2 diabetes mellitus, Cardiovascular disease, Prospective follow-up, Complications, Genetics, Case–control

## Abstract

**Background:**

Type 2 diabetes is a major healthcare problem. Glucose-, lipid-, and blood pressure-lowering strategies decrease the risk of micro- and macrovascular complications. However, a substantial residual risk remains. To unravel the etiology of type 2 diabetes and its complications, large-scale, well-phenotyped studies with prospective follow-up are needed. This is the goal of the DiaGene study. In this manuscript, we describe the design and baseline characteristics of the study.

**Methods:**

The DiaGene study is a multi-centre, prospective, extensively phenotyped type 2 diabetes cohort study with concurrent inclusion of diabetes-free individuals at baseline as controls in the city of Eindhoven, The Netherlands. We collected anthropometry, laboratory measurements, DNA material, and detailed information on medication usage, family history, lifestyle and past medical history. Furthermore, we assessed the prevalence and incidence of retinopathy, nephropathy, neuropathy, and diabetic feet in cases. Using logistic regression models, we analyzed the association of 11 well known genetic risk variants with type 2 diabetes in our study.

**Results:**

In total, 1886 patients with type 2 diabetes and 854 controls were included. Cases had worse anthropometric and metabolic profiles than controls. Patients in outpatient clinics had higher prevalence of macrovascular (41.9% vs. 34.8%; P = 0.002) and microvascular disease (63.8% vs. 20.7%) compared to patients from primary care. With the exception of the genetic variant in KCNJ11, all type 2 diabetes susceptibility variants had higher allele frequencies in subjects with type 2 diabetes than in controls.

**Conclusions:**

In our study population, considerable rates of macrovascular and microvascular complications are present despite treatment. These prevalence rates are comparable to other type 2 diabetes populations. While planning genomics, we describe that 11 well-known type 2 diabetes genetic risk variants (in TCF7L2, PPARG-P12A, KCNJ11, FTO, IGF2BP2, DUSP9, CENTD2, THADA, HHEX, CDKAL1, KCNQ1) showed similar associations compared to literature. This study is well-suited for multiple omics analyses to further elucidate disease pathophysiology. Our overall goal is to increase the understanding of the underlying mechanisms of type 2 diabetes and its complications for developing new prediction, prevention, and treatment strategies.

**Electronic supplementary material:**

The online version of this article (doi:10.1186/s13098-017-0245-x) contains supplementary material, which is available to authorized users.

## Background

Type 2 diabetes mellitus (T2DM) is a complex metabolic disease characterized by overweight, insulin resistance and beta-cell dysfunction [1–3]. Because of ageing and the rising prevalence of obesity, the incidence and prevalence of T2DM are increasing [4–7]. T2DM accounts for a large proportion of present and future health care expenditure in Western societies [5, 7, 8]. People affected by T2DM have an increased risk of cardiovascular events [9–13], and a poor prognosis after these events [14, 15]. In addition, T2DM gives rise to microvascular complications such as retinopathy, nephropathy and neuropathy [16–19]. We have collected a new large cohort of individuals with and without T2DM with prospective follow-up in the Netherlands: the DiaGene study.

The care for T2DM in the Netherlands is organized in primary care by general practitioners and at hospital-based outpatients clinics by medical specialists. This systematic care is based on local and international treatment guidelines aiming to reduce morbidity and mortality through optimal treatment of hyperglycemia and associated metabolic complications, such as dyslipidemia, vascular dysfunction and high blood pressure [20, 21]. Treatment of these components has proven to reduce the risk of cardiovascular morbidity and mortality in T2DM [22–30]. However, a substantial residual risk remains. Improving knowledge on genetic, biochemical and environmental (lifestyle and anthropometric) determinants of T2DM and its micro- and macrovascular complications can have large implications for prevention, treatment and prognosis of T2DM [2, 23, 31]. Through high throughput sequencing, about 80 common genetic variants associated with T2DM have been discovered [31, 32]. These common variants only explain 5–10% of the overall predisposition of T2DM [33]. There clearly is a need to expand these analyses to additional populations.

In this paper, we present the DiaGene study, a new, multicenter T2DM cohort study collected in the Netherlands in both primary and secondary care. The main purpose of the DiaGene study is to study the analyses of genetic, biochemical and environmental determinants of T2DM and its complications. Here we describe the characteristics of our population, the prevalence of complications and future perspectives.

## Methods

### Study design

The DiaGene study is a multicenter cohort study that was coordinated by the vascular section of internal medicine of the Erasmus Medical Center and the Diabetes subunit of the Máxima Medical Center, and collected in the city of Eindhoven, The Netherlands. Eindhoven is a medium-sized city with 170,668 adult (>21 years) inhabitants in 2011. Both hospitals in Eindhoven participated in the DiaGene study: Catharina Hospital and Máxima Medical Center. In addition, the local Primary Care Diagnostic Centre participated. Hence, virtually all diabetes patients in Eindhoven were approached for inclusion through this population-based approach. Between 2006 and 2011, physicians at all three centers included a total of 2065 patients with T2DM. Of these, 179 patients were excluded from analysis. Reasons for exclusion where: no diabetes (n = 1), Type 1 diabetes (n = 30), Maturity-Onset Diabetes of the Young (n = 4), Latent auto-immune diabetes in adults (n = 3), double inclusion (n = 77), post-pancreatitis diabetes (n = 3), refusal during study period (n = 2) and missing written informed consent (n = 59); resulting in a total of 1886 patients in the study population (Additional file 1).

The control group consisted of two groups: (1) subjects recruited via advertisement in local newspapers, and (2) subjects that where included through invitation of friends and self-reported unrelated family members of participating patients. Inclusion criteria for controls was age 55 years or older. Exclusion criteria were the presence of any kind of diabetes, use of metformin or Cushing’s disease. Subjects who were approached had at least 7 days of decision-time to fully reflect on research goals and methods using physician-provided information, before giving their written informed consent. Eventually, 904 diabetes-free subjects participated as controls. Of these, 50 were excluded from all analyses based on missing written informed consent (n = 14), double inclusion (n = 17), and suspected or confirmed diagnosis of diabetes (n = 19), resulting in a total of 854 controls included in the final population. This study was approved by the Medical Ethics Committees of the Erasmus MC, Catharina Hospital and Máxima Medical Center. Written informed consent was obtained from all participants.

### Definition of T2DM

Information on the diagnosis of T2DM was retrieved from the patient’s medical records. In accordance with American Diabetes Association—and World Health Organization—guidelines [34, 35], diabetes was defined as a fasting plasma glucose ≥7.0 mmol/L and/or a non-fasting plasma glucose level ≥11.1 mmol/L measured at least at 2 separate time points, treatment with oral glucose-lowering medication or insulin, and/or the diagnosis of T2DM as registered by a medical specialist. Persons with the diagnosis of type 1 diabetes (as derived from medical records and patient-questionnaires) or other types of diabetes mellitus were excluded from the study. Control subjects with fasting glucose ≥7.0 mmol/L or glycated hemoglobin (HbA1c) ≥47.5 mmol/mol were excluded. Information on T2DM status was checked by two investigators. If they did not reach consensus, the participant’s treating physician was consulted.

### Medical and family history

Each participant filled out an extensive questionnaire on their medical history (history of diabetes, metabolic disease, vascular disease, medication use and intoxications) and ethnicity of their parents (Additional file 2). We classified a participant to be Caucasian if both parents were reported to be Caucasian. Furthermore, the participant’s family history regarding diabetes and cardiovascular disease and medication usage was recorded through the questionnaire.

### Sample collection

A 20 cc Ethylene diamine tetra acetic (EDTA) fasting blood sample was taken from all participants. Samples were centrifuged (3000 rpm; 1800*g* for 15 min at 4 °C). Directly after centrifugation, the plasma and the buffy coat were separated and stored (at −80 °C) for DNA analysis and future measurements.

### Diabetes and complications of diabetes

Data on body mass index (BMI) (kg/m^2^) and blood pressure (mmHg) were extracted from medical records at inclusion. Similarly, laboratory results were extracted around time of inclusion and contained fasting glucose, glycated hemoglobin (HbA1c), total cholesterol, low-density lipoprotein cholesterol (LDL-cholesterol), high-density lipoprotein-cholesterol (HDL-cholesterol), triglycerides, creatinine and urinary albumin/creatinine-ratio. The majority of measurements were collected within 6 months prior to or after the actual date of inclusion. To estimate kidney function, the estimated glomerular filtration rate was calculated with the Modification of Diet in Renal Disease-formula. Information on the presence of cardiovascular disease in the patients treated in the hospital-based outpatient clinics was retrieved from their medical records. Cardiovascular disease comprised myocardial infarction, percutaneous coronary intervention/coronary arterial bypass graft (PCI/CABG), cerebrovascular accident, transient ischemic attack and peripheral arterial disease. PCI/CABG was defined as any invasive intervention to treat coronary arterial disease (PCI, CABG). Peripheral arterial disease was defined as an ankle-brachial index below 0.80 or below 0.90 with typical complaints, any intervention to treat peripheral arterial disease (supervised exercise training, stenting, bypass and percutaneous transluminal angioplasty, or the self-reported presence of intermittent claudication. Information on cardiovascular disease in patients from primary care and diabetes-free controls was based on self-reporting.

Microvascular complications were subdivided into retinopathy, nephropathy and neuropathy. Diabetic foot was additionally assessed. Retinopathy was scored according to the report of an ophthalmologist as absent or present and classified as non-proliferative, proliferative, or retinopathy treated with photo coagulation or intra-vitreal injections. Neuropathy was defined by a podotherapist, neurologist or the patients’ treating physician. Nephropathy was defined present when micro-albuminuria [Albumin/creatinine-ratio (ACR) ≥2.5 for men or ≥3.5 for women] was present at two of three consecutive measurements, or when high micro-albuminuria or macro-albuminuria was present at one measurement (ACR ≥12.5 for men or ≥17.5 for women). Diabetic foot was established by a podotherapist or physician according to the SIMM’s classification [36]. All information on laboratory data, macrovascular, and microvascular events in case and control subjects at baseline that was retrieved from medical records was separately checked by two investigators. When they did not reach consensus, the participant’s physician was consulted.

### Genotyping

DNA was isolated using the Invisorb^®^ Blood Universal Kit from Stratec Molecular (Berlin, Germany). Eleven well-known T2DM genetic risk variants were genotyped: TCF7L2(rs7903146), PPARG-P12A(rs1801282), DUSP9(rs594532), CENTD2(rs1552224), THADA(rs7578597), HHEX(rs1111875), CDKAL1(rs7754840) and KCNQ1(rs231362)which had previously been genotyped for replication in DIAGRAM [37], and KCNJ11(rs5219), IGF2BP2(rs4402960) and FTO(rs8050136). These risk variants were chosen because of their relatively large effect sizes on T2DM risk in previous studies [32, 37–42]. Genotyping was performed with TaqMan allelic discrimination assays, designed and optimized by Applied Biosystems (Foster City, CA, USA). Reactions were performed on the Taqman Prism 7900 HT platform.

### Follow-up data

Currently, we are finalizing the first collection of prospective follow-up in our study population. This encompasses all anthropometric and laboratory measurements and data on metabolic, microvascular and macrovascular complications of T2DM and enables us to perform prospective analyses.

### Statistical analysis

Continuous variables are expressed as median with interquartile range unless otherwise specified. Comparisons between groups were performed with Mann–Whitney U tests for continuous and χ^2^ tests for categorical data. Deviation from the Hardy–Weinberg equilibrium was assessed by *χ* ^2^ testing. Associations of the genotypes with T2DM were tested using logistic regression models. We have calculated interaction effects of odds ratios for T2D to compare our results with previous genetic studies according to the method of Altman et al. [43]. All models were adjusted for age and sex. Additionally, models were adjusted for center of inclusion as a categorical covariate. Cases and controls of non-Caucasian ethnicity were excluded from the genetic analyses. P values smaller than 0.05 were considered to be statistically significant. Statistical analysis was performed with SPSS-software version 22.0 (SPSS, Chicago, IL, USA).

## Results

### General characteristics

The most relevant general characteristics of the cohort are displayed in Table 1. A total of 1886 patients with T2DM and 854 diabetes-free controls were included. Of all anthropometric measurements, 90.6 and 96.1% were performed within 6 and 12 months of inclusion, respectively. Of laboratory data, 81.8 and 93.2% were measured within 6 and 12 months of inclusion, respectively. The cases and controls were of similar age. When compared to controls, cases had higher BMI [29.5 (Interquartile range (IQR) 26.4–32.7) vs. 25.5 (IQR 23.3–27.7) kg/m^2^; P < 0.001], higher HbA1c [50.8 (IQR 43.7–57.9) vs. 37.7 (IQR 36.1–39.3) mmol/mol; P < 0.001], higher creatinine [78 (IQR 66–91) vs. 72 (IQR 63–81) µmol/L; P < 0.001], higher triglycerides [1.4 (IQR 0.9–1.9) vs. 1.2 (IQR 0.9–1.5) mmol/L; P < 0.001], lower HDL-cholesterol [1.1 (IQR 0.9–1.3) vs. 1.4 (IQR 1.2–1.6) mmol/L; P < 0.001] and lower LDL-cholesterol [2.4 (0.8) vs. 3.6 (0.9) mmol/L; P < 0.001]. A larger proportion of cases had reduced estimated glomerular filtration rate (19.7% vs. 4.7%, P < 0.001) and prevalent macrovascular disease (38.0% vs 8.3%, P < 0.001) compared to diabetes-free controls. More cases had a first-degree relative with T2DM compared to controls (64.4% vs 33.3%, P < 0.001). More baseline characteristics can be found in Table 1.

**Table 1:** General baseline characteristics of participants with and without T2DM

	Cases	Controls	P value
Number of participants	1886	854	
Female sex, n (%)	874 (46.4)	511 (59.8)	<0.001
Age, year, median (IQR)	65.7 (58.5–72.9)	64.9 (60.4–69.4)	0.72
Age of onset diabetes, year, median (IQR)	55 (47–63)	N/A	N/A
Duration of diabetes, year, median (IQR)	8.1 (2.8–13.5)	N/A	N/A
BMI, kg/m^2^, median (IQR)	29.5 (26.4–32.7)	25.5 (23.3–27.7)	<0.001
HbA1c, mmol/mol, median (IQR)	50.8 (43.7–57.9)	37.7 (36.1–39.3)	<0.001
Diabetes treatment, % (n/n-available/n-missing)			
No glucose-lowering medication	19.2 (340/1772/114)	N/A	N/A
Oral glucose-lowering agent	64.3 (1140/1773/113)	N/A	N/A
Insulin	32.3 (572/1772/114)	N/A	N/A
Systolic blood pressure, mmHg, median (IQR)	140 (129–151)	137 (124–150)	<0.001
Diastolic blood pressure, mmHg, median (IQR)	78 (71–85)	82 (76–89)	<0.001
Total cholesterol, mmol/L, median (IQR)	4.2 (3.6–4.8)	5.6 (4.9–6.2)	<0.001
Triglycerides, mmol/L, median (IQR)	1.4 (0.9–2.0)	1.2 (0.9–1.5)	<0.001
HDL-cholesterol, mmol/L, median (IQR)	1.1 (0.9–1.3)	1.4 (1.2–1.6)	<0.001
LDL-cholesterol, mmol/L, median (IQR)	2.3 (1.8–2.8)	3.5 (2.9–4.1)	<0.001
Creatinine, µmol/L, median (IQR)	78 (66–91)	72 (63–81)	<0.001
eGFR < 60 mL/min, % (n/n-available/n-missing)	21.2 (372/1756/130)	5.0 (40/795/59)	<0.001
Cardiovascular disease, % (n/n-available/n-missing)			
Any macrovascular disease	38.0 (660/1738/148)	8.3 (68/824/30)	<0.001
Ischemic heart disease	28.0 (497/1778/108)	4.9 (41/842/12)	<0.001
Ischemic brain disease	12.0 (211/1757/129)	1.4 (12/840/14)	<0.001
Peripheral arterial disease	10.8 (193/1783/103)	2.2 (18/823/31)	<0.001
Microvascular diabetes complications, % (n/n-available/n-missing)			
Any microvascular disease	34.3 (561/1637/249)	N/A	N/A
Diabetic retinopathy	17.3 (308/1778/108)	N/A	N/A
Diabetic nephropathy	23.0 (387/1684/202)	N/A	N/A
Family history, % (n/n-available/n-missing)			
First-degree relative with T2DM	64.4 (1104/1714/172)	33.3 (269/809/45)	<0.001
First-degree relative with CVD	68.3 (1086/1590/296)	68.7 (519/755/99)	0.87
Any relative with early-onset CVD	45.0 (780/1732/154)	41.5 (342/825 29)	0.09
Descent			
Caucasian descent, % (n/n-available/n-missing)	91.9 (1613/1755)	96.1 (810/843/11)	<0.001
Age of death father, year, median (IQR)	73 (65–82)	75 (67–84)	<0.001
Age of death mother, year, median (IQR)	78 (70–86)	81 (73–89)	<0.001

Table 1 shows baseline characteristics of participants from the DiaGene study

*BMI* body mass index, *CVD* cardiovascular disease, *eGFR* estimated glomerular filtration rate calculated with the Modification of Diet in Renal Disease-formula, *IQR* interquartile range, *n-total* total number of participants for whom information was available, *T2DM* type 2 diabetes mellitus

### Primary care versus hospital-based outpatient clinic

Table 2 shows baseline characteristics of patients with T2DM in primary care and hospital-based outpatient clinic. Patients with T2DM from the outpatient clinic had longer median duration of diabetes compared to primary care [12.5 (IQR 7.2–17.8) vs. 4.6 (IQR 1.2–7.9) years; P < 0.001] while they were diagnosed at a younger age [50.8 (10.8) vs. 58.4 (11.3) years, P < 0.001]. At the outpatient clinic, participants had higher BMI [30.2 (IQR 26.8–33.7) vs. 29.0 (IQR 26.0–32.0) kg/m^2^; P < 0.001], HDL-cholesterol [1.2 (IQR 1.0–1.4) vs. 1.1 (IQR 0.9–1.3); P < 0.002], HbA1c [56.3 (IQR 48.1–64.5) vs. 48.6 (43.7–53.6) mmol/mol; P < 0.001] and higher creatinine [81 (67–95) vs. 76 (64–88) µmol/L; P < 0.001]. Total cholesterol [4.3 (0.9) vs 4.2 (0.9); P = 0.04] and LDL-cholesterol [2.6 (0.8) vs. 2.3 (0.8); P < 0.001] was higher in primary care patients. A larger proportion of patients with T2DM at the outpatient clinic had reduced estimated glomerular filtration rate (25.5% vs. 15.2%, P < 0.001), macrovascular disease (41.9% vs. 34.8%; P = 0.002) and microvascular disease (63.8% vs. 20.7%) compared to patients with T2DM from primary care. We could not retrieve reliable data on neuropathy nor diabetic foot in primary care population. More patients from the outpatient clinic had a first-degree relative with T2DM compared to controls (64.4% vs. 33.3%, P < 0.001).

**Table 2:** General baseline characteristics of participants with and without T2DM

	Primary care		Outpatient clinic	P value
Number of participants	1056		830	
Female sex, n (%)	494 (46.8)		380 (45.9)	0.71
Age, year, median (IQR)	65.5 (58.1–72.9)		65.9 (58.9–72.9)	0.66
Age of onset diabetes, year, median (IQR)	59 (51–67)		51 (44–59)	<0.001
Duration of diabetes, year, median (IQR)	4.5 (1.2–7.9)		12.5 (7.2–17.8)	<0.001
BMI, kg/m^2^, median (IQR)	29.0 (26.0–32.0)		30.2 (26.8–33.7)	<0.001
HbA1c, mmol/mol, median (IQR)	48.6 (43.7–53.6)		56.3 (48.1–64.5)	<0.001
Diabetes treatment, % (n/n-available/n-missing)				
No medication	24.7 (248/1004/52)		12.0 (92/768/62)	<0.001
Oral glucose-lowering agents	72.9 (732/1004/52)		53.1 (408/768/62)	<0.001
Insulin	8.6 (86/1004/52)		63.3 (486/768/62)	<0.001
Systolic blood pressure, mmHg, median (IQR)	146 (133–159)		134 (126–143)	<0.001
Diastolic blood pressure, mmHg, median (IQR)	79 (72–86)		75 (70–80)	<0.001
Total cholesterol, mmol/L, median (IQR)	4.2 (3.6–4.9)		4.1 (3.6–4.6)	0.047
Triglycerides, mmol/L, median (IQR)	1.4 (0.9–1.9)		1.5 (1.0–2.0)	0.104
HDL-cholesterol, mmol/L, median (IQR)	1.1 (0.9–1.3)		1.2 (1.0–1.4)	0.002
LDL-cholesterol, mmol/L, median (IQR)	2.5 (2.0–3.1)		2.1 (1.7–2.6)	<0.001
Creatinine, µmol/L, median (IQR)	76 (64–88)		81 (67–95)	<0.001
eGFR < 60 mL/min % (n/n available/n-missing)	16.4 (160/978/78)		27.2 (212/778/52)	<0.001
Cardiovascular disease, % (n/n-available/n-missing)				
Any macrovascular disease	34.8 (335/963/93)		41.9 (325/775/55)	0.002
Ischemic heart disease	25.2 (252/1000/56)		31.5 (245/778/52)	0.004
Ischemic brain disease	12.7 (124/973/83)		11.1 (87/784/46)	0.302
Peripheral arterial disease	9.2 (88/958/98)		12.7 (105/825/5)	0.0181
Microvascular diabetes complications, % (n/n-available/n-missing)				
Any microvascular disease	20.7 (172/830/226)		48.2 (389/807/23)	<0.001
Diabetic retinopathy	6.1 (59/962/94)		30.5 (249/816/15)	<0.001
Diabetic nephropathy	15.4 (134/868/188)		31.0 (253/816/15)	<0.001
Neuropathy	Unknown		31.2 (238/762/68)	N/A
Family history, % (n/n-available/n-missing)				
First-degree relative with T2DM	61.4 (586/955/101)		68.2 (518/759/71)	0.003
First-degree relative with CVD	67.6 (608/899/157)		69.2 (478/691/139)	0.712
Any relative with early-onset CVD	45.0 (436/968/88)		45.0 (344/764/66)	1.0
Descent				
Caucasian descent, % (n/n-available/n-missing)	90.3 (892/988/132 )		94.0 (721/767/63)	0.005
Age of death father, year, median (IQR)	73 (65–82)		73 (65–82)	0.728
Age of death mother, year, median (IQR)	79 (72–87)		77 (69–85)	0.055

Table 2 shows baseline characteristics of participants from the DiaGene study in both primary care and hospital-based outpatient clinic

*BMI* body mass index, *CVD* cardiovascular disease, *eGFR* estimated glomerular filtration rate calculated with the Modification of Diet in Renal Disease-formula, *IQR* interquartile range, *n-total* total number of participants for whom information was available, *T2DM* type 2 diabetes mellitus

### Genetics

Table 3 shows the associations of 11 well-established genetic T2DM variants in our study population. Hardy–Weinberg’s equilibrium was met for all variants. With the exception of the variant in KCNJ11, all T2DM susceptibility variants had higher allele frequencies in cases with T2DM than in controls. TCF7L2 showed the highest odds ratio for prevalent T2DM [OR 1.37 (95% CI 1.17, 1.60; P < 0.001]. These results were unaffected by additional correction for center of inclusion. After calculation of interaction effects, the associations of all genetic variants except for KCNJ11 did not significantly differ from the large scale meta-analyses of Morris et al. [44].

**Table 3:** Allele frequencies, odds ratios and 95% confidence intervals of genetic variants and risk of T2D in DiaGene, original discovery studies and most recent meta-analysis of genome-wide-association studies

Locus-marker	Risk allele/other	DiaGene		Original discovery study results			Morris et al. [52]
		Risk allele frequency case/control/total	OR (95% CI)	Ceu-hap map	OR (95% CI)	Reference original study	OR (95% CI)
CDKAL1-rs7754840	C/G	0.36/0.31/0.35	*1.23* (*1.06*–*1.44*); *P* = *0.006*	0.31	1.12 (1.08–1.16)	[38, 39, 41, 42]	1.15 (1.11–1.19)
CENTD2-rs1552224	A/C	0.86/0.86/0.86	0.95 (0.77–1.16); *P* = 0.60	0.88	1.14 (1.11–1.17)	[37]	1.13 (1.08–1.19)
DUSP9-rs5945326	G/A	0.22/0.22/0.22	1.02 (0.88–1.18); *P* = 0.77	0.12	1.27 (1.18–1.37)	[37]	N/A (on X-chromosome)
FTO-rs8050136	A/C	0.40/0.38/0.39	1.06 (0.91–1.22); *P* = 0.45	0.45	1.15 (1.09–1.22)	[41]	1.11 (1.07–1.15)
HHEX-rs1111875	C/T	0.64/0.63/0.63	1.01 (0.87–1.16); *P* = 0.95	0.56	1.13 (1.08–1.17)	[38]	1.15 (1.11–1.18)
IGFBP2-rs4402960	T/G	0.33/0.32/0.33	1.01 (0.87–1.18); *P* = 0.89	0.29	1.17 (1.10–1.25)	[41]	1.13 (1.09–1.17)
KCNJ11-rs5219	T/C	0.37/0.39/0.37	0.92 (0.80–1.07); *P* = 0.29	0.50	1.15 (1.09–1.21)	[38]	1.08 (1.05–1.12)
KCNQ1-rs231362	G/A	0.53/0.49/0.52	*1.16* (*1.00*–*1.34*); *P* = *0.04* *	0.52	1.08 (1.06–1.10)	[40]	1.11 (1.07–1.16)*
PPARG-P12A-rs1801282	C/G	0.89/0.88/0.89	1.13 (0.91–1.42); *P* = 0.27	0.92	1.14 (1.08–1.20)	[38, 39, 42]	1.16 (1.11–1.22)
TCF7L2 rs7903146	T/C	0.36/0.28/0.34	*1.37* (*1.17*–*1.60*); P < *0.001*	0.25	1.37 (1.28–1.47)	[41]	1.40 (1.35–1.46)
THADA rs7578597	T/C	0.91/0.88/0.90	*1.36* (*1.08*–*1.71*); *P* = *0.01*	0.92	1.15 (1.10–1.20)	[41]	1.14 (1.08–1.22)

Table shows odds ratios of association with type 2 diabetes for different known risk alleles tested in our study population. Logistic regression analysis is age, sex and center of inclusion-adjusted

Significant odd ratios of association with type 2 diabetes are displayed in italic

*CEU* Caucasian, *OR* odds-ratio, *CI* confidence interval

* Statistically significant difference of odds ratio in association of genetic variant with T2DM when compared to Morris et al. [52]

## Discussion

In this manuscript, we present the baseline characteristics and future perspectives of the DiaGene study, a new multi-centre cohort study with prospective follow-up on biochemical and genetic determinants of T2DM and its complications. We show that the population is representing both primary and secondary care and that despite treatment, considerable rates of macrovascular and microvascular complications are present. To further elucidate determinants of T2DM and its complications, multi-layer omics and prospective analyses will be of great value. Our study offers excellent opportunities to perform these analyses.

In the Netherlands, primary care practices are led by general practitioners, who are easily accessible and offer essential family medicine. Outpatient clinics of hospitals provide specialized care and require referral by the general practitioner for reimbursement by insurance companies. Therefore, complex and more severely affected patients will be referred to the hospital-based outpatient clinics. This is reflected in the higher prevalence of micro- and macrovascular complications at the outpatient clinics in our population.

The risk of microvascular disease can be reduced substantially by glycemic control and general measures to prevent cardiovascular disease such as lifestyle, blood pressure and lipid optimization [22, 23, 25]. Rates of microvascular disease in our study at baseline were 17.3, 23.0 and 31% for retinopathy, nephropathy and neuropathy, respectively. This incidence of retinopathy in T2DM is comparable to a report from the Dutch National Institute for Public Health and the Environment [45] and in line with a worldwide meta-analyses for diabetes with a duration of less than 10 years [46], but higher than in a screening study for T2DM from the Netherlands [47]. In the latter study, the duration of T2DM was short and this probably explains the difference. For nephropathy, our rate is slightly lower than in the United Kingdom Prospective Diabetes Study (25%), also probably because of shorter follow-up [16]. Our primary care population appeared to have lower rates of nephropathy compared to studies on prevalent diabetes and newly diagnosed diabetes in patients of general practitioners in the Netherlands [47, 48]. Although the single urinary measurement-based prevalence rates in the latter could be an explanation for this discrepancy. The percentage of patients with T2DM and neuropathy in our population (31%) is lower compared to a prospective study (50%) with 25 years of follow-up from diagnosis [19] and comparable to a cross-sectional study on peripheral neuropathy in the United Kingdom [49].

The risk of macrovascular disease in T2DM can be successfully reduced by applying lifestyle interventions, lipid lowering therapies and antihypertensive treatment. The relationship with glycemic control is more complex. Even though glycemic control epidemiologically is strongly related to cardiovascular disease in T2DM, interventions applying strict glycemic control were unsuccessful [22, 50] or even showed adverse effects [51]. Macrovascular disease rates in our population with T2DM is comparable to previous reports in the Netherlands [45, 52], but lower than in an interview-based study in diabetes patients in the USA [53]. Our population is on average 5 years older than the patients in this American study, and also contains a significant proportion of patients from outpatient clinics having further progressed disease.

T2DM and its complications are multifactorial in their pathophysiology’s. Genetics, epigenetics, biological mechanisms and environmental factors are probably interacting at multiple levels. Therefore a pathway-based approach in well-defined cohorts is needed, supported by full use of information technology. High throughput research has been mainly focused on genome wide genetic associations. This has elucidated interesting associations. Yet the results only explain disease susceptibility to a small extent [31, 33]. We are planning to perform genome-wide association analysis in the near future. The quality control of this future genomic work will include analyses of the genetic-based ethnic background to definitively determine population sub-structures. Here, we restricted our analyses of well-known genetic T2DM risk variants to the sub-group of self-reported Caucasians. These DNA polymorphisms showed similar associations in our mainly Caucasian population as in previous extensive meta-analyses: most genetic variants had similar direction of their associations as earlier reported and for TCF7L2, THADA, KCNQ1 and CDKAL1 this was significant [32, 44, 54]. KCNJ11 and CENTD2 showed a slight but not statistically significant opposite association to what has previously described, with estimates close to 1 and confidence intervals embracing the estimates from literature [32]. Except for KCNJ11, all genetic variants had non-significant interaction effects for odds ratios of T2DM-risk variants compared to the latest meta-analysis [44]. The significant difference for KCNJ11 can be an effect of population-specific variance, differences in environmental factors, age or interactions of these factors with the genetic variant [44].

To study the aetiology of T2DM and its complications we need well phenotyped cohorts with prospective follow-up. Our population has these characteristics. We therefore plan to analyse several omics layers for their associations with T2DM and its complications. We are currently measuring total N-glycomics with matrix-assisted laser desorption–ionization-time of flight (MALDI-TOF), matrix-assisted laser desorption–ionization-Fourier transform ion cyclotron resonance mass spectrometry (MALDI-FTICR) [55] and IgG-glycomics with ultra-performance liquid chromatography [56]. In the near future, we aim to include lipidomics, with a focus on lipoprotein(a), metabolomics, and proteomics. Also we plan to perform genomics using the Illumina chip, for mendelian randomization and multilayer interaction analyses. The overall goal being to elucidate new pathophysiological pathways for prediction, prevention and treatment of T2DM.

Although we have performed our study with precision, we need to consider a number of limitations. A large majority of our population is of self-reported Caucasian ethnicity, which limits extending conclusions from our analyses to non-Caucasian populations. However, it also makes our analyses less vulnerable to genetic population stratification bias. Self-reported Caucasian mono-ethnicity in two generations results in a very limited risk of misclassifying genetic admixtures [57, 58]. In addition, a small proportion of diabetes-free subjects where recruited by asking T2DM subjects to invite unrelated family members and friends. Hence, absence of family ties was self-reported, with a small possibility of hidden relatedness. In the near future, we will perform genome-wide association analysis, which will allow us to perform formal quality control and accurately account for hidden relatedness and genetic population stratification bias [59]. Another limitation of this study was our inability to retrieve information on neuropathy in primary care setting. Conclusions on neuropathy are therefore restricted to the secondary care setting. We have made extensive efforts to optimise the reliability of our data by having two independent investigators collect the data and reach consensus. This means we did have to rely on common clinical practice and adequate record keeping in primary and secondary care. For macrovascular events in primary care we had to rely on self-reported data. For validation, we have therefore checked self-reported myocardial infarction data from hospital-based participants and found that in only 6.0% of participants with self-reported myocardial infarction this diagnosis was not confirmed in hospital data. These events have therefore been scored as missing. Underestimation of the incidence of myocardial infarction based on hospital discharge data has however been described before [60]. And although the questionnaire on lifestyle, medication, clinical events and family history was straight-forward and easy to use, it is not an externally validated questionnaire. At last, our preliminary genetic results had approximately 10% missing values. We are currently collecting additional samples from the participants whose DNA was not available at the time of the current genetic analysis to improve our genetic analysis. Further strengths of our study are the meticulous hands-on medical file review for each patient by two separate physicians, which produced high-quality data that enable us to research both T2DM itself as well as its complications in great detail. Currently, we are finalizing the first collection prospective follow up on all T2DM complications. The prospective cohort setting with concurrent inclusion of diabetes-free individuals at baseline, will allow us to perform cross-sectional and prospective end-point analyses to study aetiology and progression of type 2 diabetes and its complications.

## Conclusion

In conclusion, this manuscript describes the design and baseline characteristics of the DiaGene study, a large multi-centre prospective follow-up cohort study on environmental, biochemical and genetic risk factors of T2DM and related vascular complications. By studying both clinical and complex biochemical parameters with a current focus on glycomics, genomics and lipidomics, the DiaGene study aims to contribute to the pathophysiological understanding of T2DM and all its vascular complications in a prospective case–control setting.

## Additional files

**Additional file 1.** Flow chart of the inclusion of cases and controls.

**Additional file 2.** Questionnaire used in Diagene study.

## Abbreviations

ACR: albumin/creatinine-ratio
BMI: body mass index
CABG: coronary arterial bypass graft
DNA: deoxyribonucleic acid
EDTA: ethylene diamine tetra acetic
HbA1c: glycated hemoglobin
HDL: high-density lipoprotein
IQR: interquartile range
LDL: low-density lipoprotein
MALDI-FTICR: matrix-assisted laser desorption–ionization-Fourier transform ion cyclotron resonance mass spectrometry
MALDI-TOF: matrix-assisted laser desorption–ionization-time of flight
PCI: percutaneous coronary intervention
T2DM: type 2 diabetes mellitus
