# Supplementary material for: Introduction of the DiaGene study: clinical characteristics, pathophysiology and determinants of vascular complications of type 2 diabetes
Source: Diabetol Metab Syndr. 2017 Jun 19;9:47. doi: 10.1186/s13098-017-0245-x (PMC5477157; doi:10.1186/s13098-017-0245-x)
Supplement: Supplementary file 1 — Additional file 1. Flow chart of the inclusion of cases and controls. [file 13098_2017_245_MOESM1_ESM.docx]

**Additional file 1:** Flow chart of the inclusion of cases and controls

**
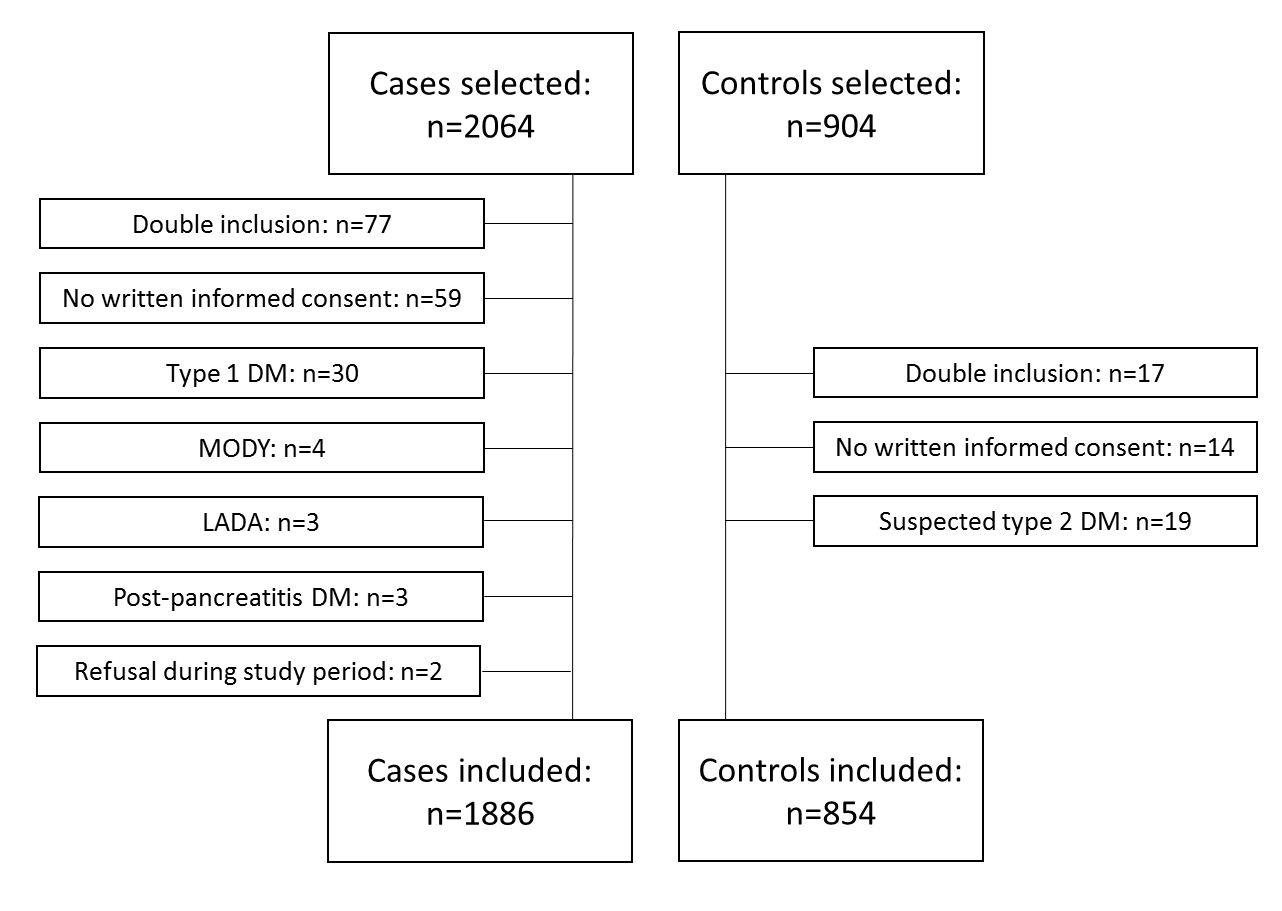
**

DM**,** diabetes mellitus; MODY, Maturity-Onset Diabetes of the Young; Latent Auto-immune Diabetes of the Adult
