# Supplementary material for: Introduction of the DiaGene study: clinical characteristics, pathophysiology and determinants of vascular complications of type 2 diabetes
Source: Diabetol Metab Syndr. 2017 Jun 19;9:47. doi: 10.1186/s13098-017-0245-x (PMC5477157; doi:10.1186/s13098-017-0245-x)
Supplement: Supplementary file 2 — Additional file 2. Questionnaire used in Diagene study. [file 13098_2017_245_MOESM2_ESM.docx]

**Additional file 2: Questionnaire used in Diagene study**

1. **Personal data**

Name …………………………………………………………

Telephone number:………………………………. …………………………….

Date of birth: ………………………………………………………………

Profession: ………………………………………………………………………

**Questionnaire Diagene**

**1. Personal information**

**-** What is your sex? 🞏 Male

🞏 Female

- What is your age? Year

1. **Medical history**

- Do you have diabetes?

- Do you have an elevated cholesterol? 🞏 Yes

🞏 No

If so, what was the highest value : *(Fill in your cholesterol)*

Have you ever had any of the following medical conditions?

- Heart disease (myocardial infarction/ angina pectoris?) 🞏 yes

🞏 NO

- Stroke ? (Brain infarction) 🞏 Yes

🞏 No

- Elevated blood pressure? (hypertension) 🞏 Yes

🞏 No

- Peripheral Arterial Disease? (claudicatio intermittens) 🞏 Yes

🞏 No

- Thyroid ilness 🞏 Yes

🞏 No

- Have you ever had surgery? 🞏 Yes

🞏 No

If you indeed had surgery, what was done and when?

| **Year** | Operation performed |
| --- | --- |
|  |  |
|  |  |
|  |  |
|  |  |
|  |  |
|  |  |
|  |  |

**3.FAMILY HISTORY**

Are there any of your own family members (your own relatives) that have had a myocardial infarction or stroke at a young age? (Before the age of 60) 🞏 Yes

🞏 No

Which persons in your family have heart problems or vascular disease at what age? (Pleas consult your family)

- My father 🞏 Yes 🡪 at age:

🞏 No

- My mother 🞏 Yes 🡪 at age:

🞏 No

- My brother/sister 🞏 Yes 🡪 at age:

🞏 No

- A grandparent 🞏 Yes 🡪 at age:

🞏 No

- An uncle/aunt 🞏 Yes 🡪 at age:

🞏 No

- A cousin 🞏 Yes 🡪 at age:

🞏 No

- Other family, namely: op at age:

**-** Do you have brothers or sisters with diabetes? 🞏 Yes

🞏 No

**Your father**:

- What is the year of birth of your father? *(Fill in a year)*

- If he is deceased, what was his year of death?  *(Fill in a year)*

- If he is deceased, what was the cause of death?

- Did he or does he have diabetes? 🞏 Yes

🞏 No

- What was/is his place of birth?

- What was his ethnicity? 🞏 Caucasian 🞏 Asian

(Please choose one) 🞏 African 🞏 Admixture

🞏 Hindustan 🞏 Other, namely

**Your mother:**

- What is the year of birth of your mother? ( *(Fill in a year)*

- If she is deceased, what was his year of death? *(Fill in a year)*

- If she is deceased, what was the cause of death?

- Did she or does she have diabetes? 🞏 Yes

🞏 No

- What was/is her place of birth?

- What was her ethnicity? 🞏 Caucasian 🞏 Asian

(Please choose one) 🞏 African 🞏 Admixture

🞏 Hindustan 🞏 Other, namely

**4. MEDICATION USAGE**

Do you use medication? 🞏 Yes

🞏 No

(Please fill in as complete as possible, including contraceptives, warfarin, pain killers and insulin with dosage)

| Name of medication | Number a day | Dosage (mg) | Since when? |
| --- | --- | --- | --- |
|  |  |  |  |
|  |  |  |  |
|  |  |  |  |
|  |  |  |  |
|  |  |  |  |
|  |  |  |  |
|  |  |  |  |
|  |  |  |  |
|  |  |  |  |
|  |  |  |  |
|  |  |  |  |
|  |  |  |  |
|  |  |  |  |

Pay attention! Questionnaire continues on the backside

**5. Lifestyle**

- Do you smoke? 🞏 Yes

🞏 No

- If yes, since when?  *(Fill in a year)*

- What do you smoke? a) Cigarettes 🞏 Yes c) Shag 🞏 Yes

🞏 No 🞏 No

b) Cigars 🞏 Yes d) Pipe 🞏 Yes

🞏 No 🞏 No

- How many a day approximately? *(Fill in number)*

- Have you ever smoked previously? 🞏 Yes

🞏 No

- If yes, how many a day approximately? *(Fill in number)*

- If yes, from when to when? From To  *(Fill in year)*

Or how many year? *(Fill in number of years)*

- Do you use alcohol? 🞏 Yes

🞏 No

- If yes, what kind of alcohol?

Liquor 🞏 Yes

🞏 No

Beer 🞏 Yes

🞏 No

Wine 🞏 Yes

🞏 No

Other, namely:

- If yes, how many glasses a day? *(number of glasses)*
